# *Qigong* as a Traditional Vegetative Biofeedback Therapy: Long-Term Conditioning of Physiological Mind-Body Effects

**DOI:** 10.1155/2015/531789

**Published:** 2015-06-07

**Authors:** Luís Carlos Matos, Cláudia Maria Sousa, Mário Gonçalves, Joaquim Gabriel, Jorge Machado, Henry Johannes Greten

**Affiliations:** ^1^Instituto de Ciências Biomédicas Abel Salazar (ICBAS), Universidade do Porto, Largo Professor Abel Salazar 2, 4099-030 Porto, Portugal; ^2^German Society of Traditional Chinese Medicine, Karlsruher Straße 12, 69126 Heidelberg, Germany; ^3^Heidelberg School of Chinese Medicine, Karlsruher Straße 12, 69126 Heidelberg, Germany; ^4^Departamento de Engenharia Mecânica, Faculdade de Engenharia da Universidade do Porto, Rua Dr. Roberto Frias, s/n, 4200-465 Porto, Portugal; ^5^Porto Biomechanics Laboratory (LABIOMEP), University of Porto, Rua Dr. Plácido Costa 91, 4200 450 Porto, Portugal

## Abstract

A contemporary understanding of Chinese Medicine (CM) regards CM diagnosis as a functional vegetative state that may be treated by vegetative reflex therapies such as acupuncture. Within this context, traditional mind-body exercises such as *Qigong* can be understood as an attempt to enhance physiological proprioception, by combining a special state of “awareness” with posture, movement, and breath control. We have formerly trained young auditing flutists in “White Ball” *Qigong* to minimize anxiety-induced cold hands and lower anxiety-induced heart rate. Functional changes occurred 2–5 min after training and were observed over the whole training program, allowing the children to control their symptoms. In our current work, we report that warm fingers and calm hearts could be induced by the children even without *Qigong* exercises. Thus, these positive changes once induced and “conditioned” vegetatively were stable after weeks of training. This may show the mechanism by which *Qigong* acts as a therapeutic measure in disease: positive vegetative pathways may be activated instead of dysfunctional functional patterns. The positive vegetative patterns then may be available in critical stressful situations. *Qigong* exercise programs may therefore be understood as an ancient vegetative biofeedback exercise inducing positive vegetative functions which are added to the individual reactive repertoire.

## 1. Introduction

The contemporary understanding of the diagnosis in Traditional Chinese Medicine (TCM) considers TCM as a traditional model of vegetative system biology with the purpose of a systemic therapeutic approach [1–8]. Within this concept,* Qigong* is understood as a traditional vegetative biofeedback therapy consisting of concentrative motion and postures combined with breathing exercises. Actually the “*qi* activation” is achieved by breath control and a special mental state of “awareness” [9, 10], thereby improving and strengthening the overall state of vegetative regulation (homeostasis) [1, 11–15].

Several studies on* Qigong*-related effects concerning physiological processes and variables have been published elsewhere. Actually some of these studies point to significant changes on parameters such as the blood pressure, heart rate and variability, decrease of plasma triglycerides, total cholesterol and low density lipoprotein (LDL) cholesterol, increase of HDL cholesterol, skin temperature, and improvement in lung functions such as the increment in forced expiratory volume and the reduction in the number of exacerbations, between others [16–22].

One of the prime benefits of* Qigong *seems to be stress reduction, and one of the main concepts of this practice is to use the mind to guide activation and deactivation patterns by imagination. Excessive stress may have a negative impact on the health state of a person and may be associated with an increase of anxiety, psychological disorders, and functional impairments of organs within the body [11]. It was shown that* Qigong* training may reduce emotional exhaustion and depersonalisation and even improves anxiety and reinforce attention and effectiveness in high school students [23–34].

Although in some European countries* Qigong* is considered and supported by the health systems as a preventive measure, training programs for children are not widely spread. As this is possibly due to a lack of data, we were interested in a further understanding of the modes of action of* Qigong*. We chose the so-called “White Ball” exercise system, as it takes 5 min to do it and can be integrated at work or at school [9, 10].

In its traditional understanding, TCM holds that “*qi*,” a functional power in the body, may be seen in functional physical signs. These are related to measurable physiological processes and aspects such as the increase of the peripheral microcirculation, thus the increase of the skin temperature and the changes on acupoints electrical potential and resistance and even on the surrounding biomagnetic field [24, 35–37]. Objective detection methods to evaluate the physical effects of* Qigong* therefore are an important prerequisite for the development of clinical study designs. Some authors have reported significant changes in the intensity and frequency of the infrared radiation emitted from the hands of* Qigong* practitioners, as well as detection of dynamic changes of temperature by thermography [24, 38, 39]. Qin et al. (1997) suggested that infrared thermography could be used to measure the dynamic changes of temperature in the hands and arms during* Qigong* practice [39].

We had previously refined this method and shown that young flutists, suffering from cold hands and elevated heart rate before auditions, would show elevation of hands temperature and reduction of anxiety-induced heart rate after the* Qigong* exercises and along the training program. We were now interested whether this effect was only to be seen while training was going on or whether* Qigong* exercises can result in stable changes of the functional vegetative repertoire, which we could even call a remodelling of vegetative functions and pathways. In this sense, stable changes of vegetative functional reactions would explain long-term effects of* Qigong* by the remodelling of vegetative functional patterns. It would also explain some of the positive long-term emotional changes, as emotionality and body experience are understood as interrelated [5, 40].

## 2. Materials and Methods

### 2.1. Sampling and Study Design

A group of seven healthy Caucasian volunteer children, aged between 10 and 12 years, six females and one male, students of flute from a local music academy, and without any previous experience in* Qigong,* were examined for* Qigong*-related effects against performance-related anxiety in well-controlled conditions [25]. In addition to the physiological measurements of that study, we could examine the effects of the exercises by thermography of the hands prior to and after a seven-week* Qigong* training program. Control experiments were conducted with the same individuals, so each one performed two consecutive measurements, the baseline or control and the* Qigong* exercise. In these two assays, the adopted physical posture was the same; however, the intention, mind focus, or the achievement of a “special mental state of awareness” was completely different.

### 2.2. Infrared Thermography

Experiments were performed at a mean room temperature of 20°C measured with a type K thermocouple connected to a Labfacility digital thermometer, model 2000L. An Infrared camera from FLIR, model A325 (sensibility < 0.07°C; precision ±2%), was used and supported by a tripod, placed 2 metres away from the target. Capture and image analysis were carried out with the program ThermaCAM Researcher Pro 2.9 from FLIR Systems, and the recording frequency was one photo every ten seconds.

### 2.3. *Qigong* Posture and Training

The* Qigong* exercise selected for this study was the “White Ball” standing exercise according to the Heidelberg Model of TCM as described in detail elsewhere [10]. In brief, the exercise chosen from this system includes a nondynamic basic* Qigong* posture, similar to the* Wu Chi* posture in the* Zhan Zhuang* system [41–43], minimizing the effects of physical movement. In the exercise, the imagination of holding the ball in front of the abdomen (so-called lower* Dantian*) is used to induce a sensation traditionally referred to as “*qi*” sensation, similar to “deqi” sensation observed in acupuncture. Children were instructed to do the exercise daily for seven weeks. They had accompanied training for 30 minutes with an experienced* Qigong* practitioner twice a week.

### 2.4. Statistical Analysis

The following variables were considered in the thermography analysis: final temperature on the tip of the middle finger (FT TMF); activation time (AT: time to increase 1°C on the tip of the middle finger); final temperature on* Láogōng*, Pc8 (FT Pc8); heating rate (HeatR—°Cs^−1^).

Pearson correlation analysis and principal components analysis (PCA) were performed to detect structure in the relationships between variables. Eigenvalues were observed and two factors were enough to explain almost all the variabilities. Results are shown as figures and tables. Pearson correlation analysis and PCA were performed with Statistica for Windows release 6.0.

## 3. Results and Discussion

**Figure 1:**
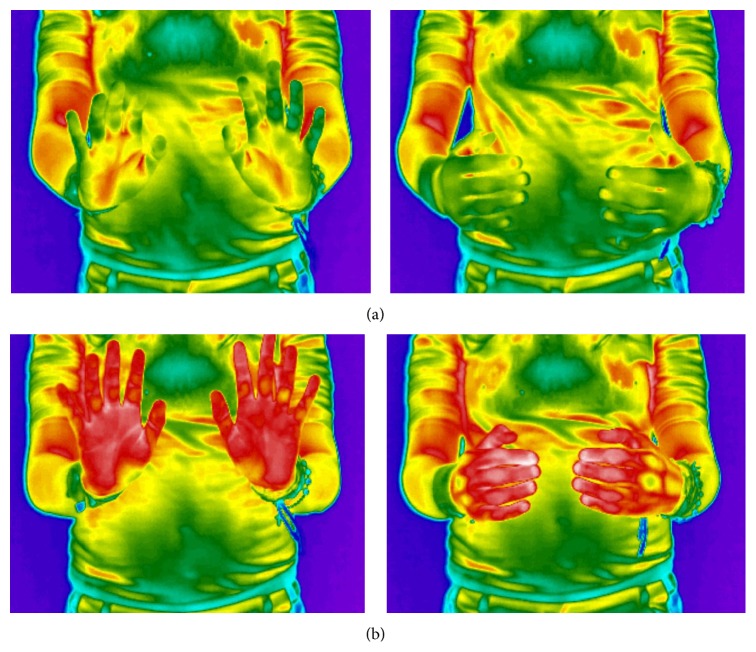
Thermograms of the* Qigong* exercise ((a) before; (b) after).

In brief, thermography indicated changes of local microcirculation during the exercises as shown in Figure 1.

**Figure 2:**
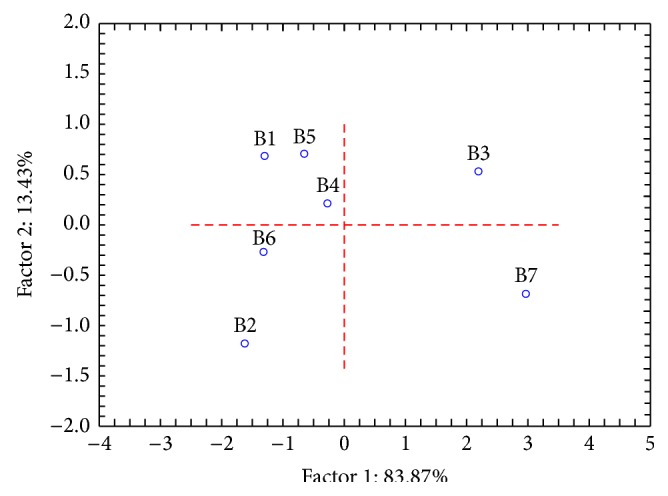
Principal components analysis (PCA) concerning the variables FT TMF, AT, FT Pc8, and HeatR at the beginning of the* Qigong* program.

**Figure 3:**
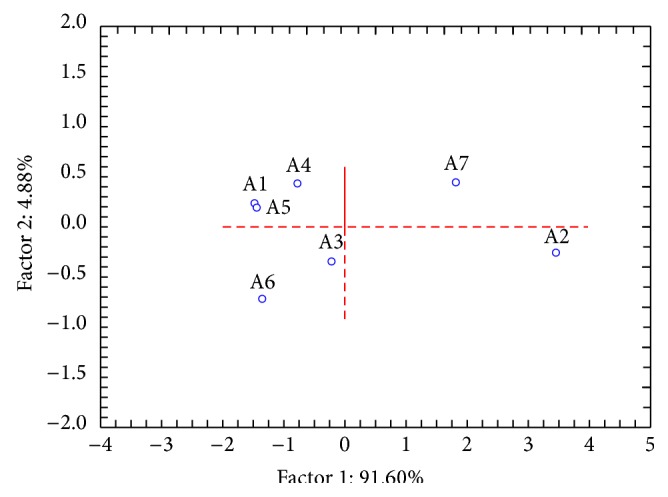
Principal components analysis (PCA) concerning the variables FT TMF, AT, FT Pc8, and HeatR at end of the* Qigong* program.

**Figure 4:**
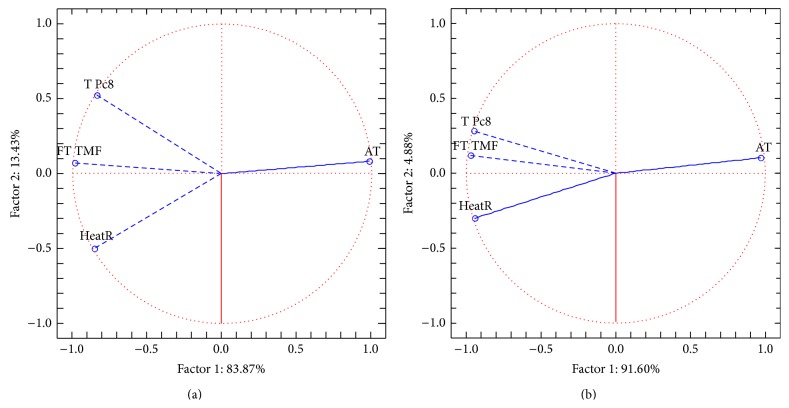
PCA variables projection on the graphical plane: (a) before and (b) after the* Qigong* program.

Statistically significant changes in temperature measured by thermography occurred during the exercises and at the beginning and at the endpoint of the observation interval. Highly significant differences (*P* < 0.01) were obtained when comparing the first and second assays with the respective baselines. Moreover, when comparing the first with the second assays no statistically significant differences were obtained (*P* = 0.364). This could be an indicator that this group of children quickly developed the capacity of vegetative activation through* Qigong* practice being able to minimize anxiety-induced cold hands in a short period of time and on demand as a part of the child reactive repertoire. Furthermore, there was an improvement on the performance of almost every child along the* Qigong* program, with a tendency to a homogeneous response on the measured vegetative effects, as can be seen when comparing the PCA results shown in Figures 2 and 3. On the mentioned figures, dots are plotted according to the PCA scores related to the variables (Figure 4) and represent each child, before (b) and after (a) the* Qigong* program. Moreover, dots plotted on Figure 3 are close to each other, thus indicating a group tendency that reflects the vegetative conditioning of positive physiological effects within the program.

**Figure 5:**
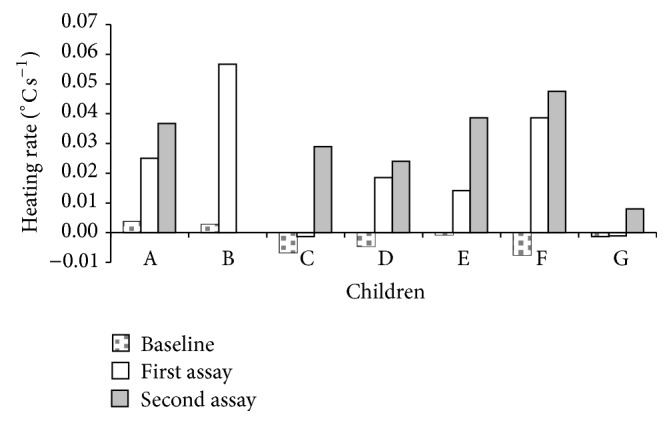
Heating rate for each child (A to G) in the baseline assay and at the beginning and end of the* Qigong* training program.

As can be seen in Figure 5, with exception of child B, there was a general noticeable increase of the heating rate at the end of the* Qigong* training program. This means that children developed the ability to quickly activate and increase the hands microcirculation, thus increasing the hands temperature in a shorter period of time.

The time until physiological activation was less than 2 minutes in all examined cases. The results indicate that, like in other* Qigong* exercises, the system of the “White Ball” can change the temperature of the skin as shown by thermography. Unlike other “*qi*” exercise cycles in TCM, the “White Ball” system is only a short exercise of approximately 5 minutes. The short duration nature of this* Qigong* exercise can be confirmed by the activation time of less than 2 minutes in all tested individuals.

Heart rate results point to a significant decrease of this variable along the* Qigong* program, thus indicating a higher level of relaxation and a lower level of anxiety. Actually the mean heart rate at the beginning of the program was 102.9 beats per minute, with a standard deviation of 20.5 beats per minute and at the end these values were 92.0 and 17.2, respectively.

As can be seen in Table 1, the heating rate correlates negatively with the heart rate, meaning that the heart rate tends to decrease as the faster hands temperature gets higher. Oppositely, the final temperature on the tip of the middle finger (FT TMF) correlates positively with the heating rate, thus indicating that the faster the heating rate, the higher the FT TMF. In both cases, the correlation coefficient *r* is higher for the results of the second assay, pointing to an improvement of the* Qigong* group.

## 4. Conclusions

Our findings show that children quickly learned this* Qigong *system with obvious development of individual vegetative skills and the reduction of anxiety-induced effects. These effects were stable after weeks of training, allowing for playing the flute with warm fingers and reduction of the anxiety-reduced elevation of the heart rate even without prior application of* Qigong *exercises. It seems that these positive vegetative changes in the behavioural pattern appear to be naturally available on demand in critical stressful situations as a part of the child reactive behavioural repertoire. We consider this to be an effect by conditioning a vegetative new pattern. Therefore, our data suggest that, by the exercise, a change in the functional vegetative pattern is first elicited. This may be then engrammed and conditioned to be elicited by another stimulus, in this case the context of flute and audition. Having to audit then elicits a functional pattern that is free from anxiety-induced manifestations of the vegetative system.

Overcoming the dysfunctional and disturbing effects of individual challenges in life may be considered to be a necessity for the prevention of a number of constellations like blackouts in examinations, failure in professional presentations, panic attacks, and states of anxiety and burn-out. Our data suggest that* Qigong*, if correctly understood and practised, may be a useful tool in this context.

Our data may also be helpful in the demystification of Chinese Medicine. Thermography allows visualizing the effects of the microcirculation on the hands temperature during* Qigong *practice. Actually, TCM holds that the “mind” guides the “*qi*” which guides the “*xue*” (blood).

The Heidelberg model of TCM sees strong analogies of the effects of “*xue*” as described by the classical scriptures with the clinical effects of microcirculation in Western medicine. Therefore, “*qi*,” translated by this model as a vegetative functional capacity, guides and steers the microcirculation. In other words, this old phrase from classical scriptures can be demystified as “the mind” (imagination and awareness) can guide and therefore activate vegetative capacities, which in return lead to changes in microcirculation.

We believe that such demystifying approaches to the corpus medicus of Chinese Medicine may be helpful to objectify its vegetative mechanisms underlying* Qigong* therapy and acupuncture. These exercises should be further explored and demystified as an ancient form of vegetative biofeedback therapy with long clinical experience.

The “White Ball” system is a simple, short duration* Qigong *exercise, easy to learn, that does not require much space, with positive effects on anxiety and stress management. It could be a powerful tool in the management of these disorders even when used in a classroom, at work, or elsewhere. As the conditioning effect may be more pronounced in the developing nervous system, these helpful patterns may be especially worthwhile and effective in children. Therefore, the supposed mechanisms of conditioning positive vegetative behavioural patterns suggest the application of these exercises especially at a young age.

## Figures and Tables

**Table 1:** Correlation between the heating rate (HeatR) and the heart rate (HR) and final temperature on the tip of the middle finger (FT TMF) at the beginning and ending of the *Qigong* training program.

Variable	HR	FT TMF
HeatR beginning	*r* = −0.423	*r* = 0.764
	*P* = 0.345	*P* = 0.045

HeatR ending	*r* = −0.625	*r* = 0.878
	*P* = 0.133	*P* = 0.009
